# Electrochemotherapy as Promising Treatment Option in Rare Recurrent Cutaneous Neoplasm of the Scalp: Case Report of an Elderly Patient

**DOI:** 10.1155/2019/2507642

**Published:** 2019-03-28

**Authors:** Edoardo Crimini, Michela Roberto, Valter Degli Effetti, Paolo Marchetti, Andrea Botticelli, Francesca Matilde Schipilliti, Giulia Arrivi, Federica Mazzuca

**Affiliations:** ^1^Medical Oncology Unit, S Andrea Hospital, Sapienza University of Rome, Rome, Italy; ^2^Plastic Surgery Unit, Paideia Clinic, Rome, Italy

## Abstract

**Background:**

Atypical fibroxanthoma (AFX) is a tumor that commonly presents on the head or neck in older individuals. Making a definitive diagnosis of AFX is challenging, and frequently, it is hard to distinguish from pleomorphic dermal sarcoma (PDS). There are no clear recommendations regarding the treatment of AFX, but an extensive surgery is actually considered the best option. Electrochemotherapy (ECT) is a novel therapeutic modality of local treatment in which the application of electrical pulses, enhancing cell membrane permeability, allows greater intracellular accumulation of chemotherapy drugs in the skin or subcutaneous tumors.

**Case Report:**

We report a case of a 78-year-old male affected by a red, ulcerative, dermal, scalp nodule, which was treated with ECT with a complete clinical response. We have also reported literature data on this topic.

**Results:**

In this case, ECT showed to be an effective and safe treatment for recurrent neoplasms of the head and neck, considering the complete response obtained and the absence of disease relapse after two years.

**Conclusion:**

To the best of our knowledge, this is the first case report that shows great clinical results using ECT after surgery in relapsed AFX/PDS. However, more studies are needed to confirm our results.

## 1. Introduction

Electrochemotherapy (ECT) is the most developed electroporation-based cancer treatment, and it demonstrated to be highly effective, with complete response rates between 60% and 70% and objective response (complete and partial response) rates of 80% in primary or relapsed cutaneous tumors [1, 2]. This technique is based on the principle of electroporation (EP), the temporary increasing of cell membrane permeability that occurs when short high-voltage electric pulses are applied that allows a better penetration in the cells of hydrophilic drugs, which otherwise would not pass the membrane. The chemotherapeutic agents most commonly used in association with ECT are bleomycin and cisplatin, both administrable systemically or intratumorally. ECT is primarily indicated in melanoma, Kaposi sarcoma, and cutaneous or subcutaneous metastases. Recent studies have focused on its role in the treatment of nonmelanoma skin tumors, especially basal cell carcinoma (BCC) and squamous cell carcinoma (SCC) of the head and neck [3–7]. Moreover, a meta-analysis showed that ECT is safe and cost-efficient as well as suitable for repetitive treatment [8]. Therefore, investigating the role of ECT for the treatment of atypical, aggressive, cutaneous tumors has gained a great deal of attention in the scientific community. In this article, with approval of the patient and review board of the Department of Clinical and Molecular Medicine of the “Sapienza” University of Rome, we report a case of atypical fibroxanthoma/polymorphic dermal sarcoma of the scalp, treated with ECT, resulting in long-term complete response, thus suggesting a possible new treatment opportunity for this type of cutaneous cancers.

## 2. Case Report

In February 2015, a 78-year-old white male was referred to us for the occurrence of a solitary rapidly growing nodule on the scalp. The nodule measured 3 cm in diameter. Clinical examination revealed a subcutaneous, bright red, ulcerated, dome-shaped lesion with irregular margins. The patient underwent an intervention of surgical excision, covering the substance loss with a skin graft from the left inguinal region. Subsequently, accurate haemostasis and suture were performed, and a compressive medication was applied. The histopathology described an ulcerated undifferentiated malignant neoplasm consisting of cells of different dimension and high mitotic index. The immunohistochemistry showed a weak positivity for S-100 and HMB45, negativity for CK, and moderate positivity for CD68. The surgical margins were clear and the diagnosis was atypical fibroxanthoma.

### 2.1. Follow-Up and Pathology Revision

**Figure 1:**
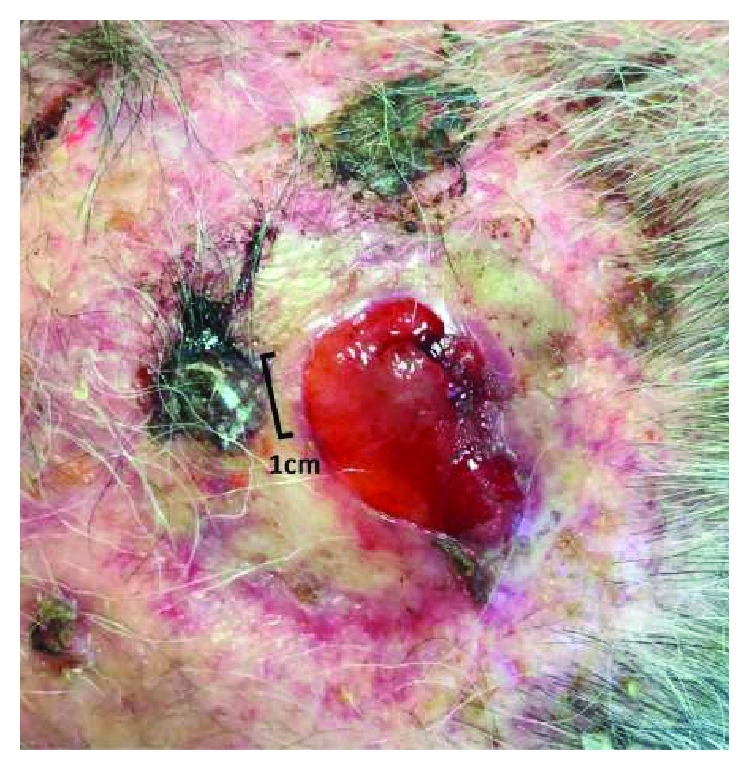
Recurrent lesion before ECT treatment.

After a month from the surgery, in May 2015, a new nodule appeared in correspondence of the graft, suggesting a recurrence (Figure 1). Considering disease relapse and the rarity of atypical fibroxanthoma, a histopathological revision of the case was requested. The second pathology report described a subcutaneous malignant neoplasm with spindle cells and pleomorphic epithelioid cells, with necrosis and atypical mitosis. De novo immunohistochemistry showed positivity for S-100 and ML actine, focal for HMB45 and MART-1, negativity for P63. Immunohistochemistry revision revealed negativity for CD68 and focal positivity for pan-CK and HMB45. This analysis suggested a diagnosis of undifferentiated pleomorphic sarcoma, but it has not excluded an acromial melanoma with aberrant ML actine expression.

### 2.2. ECT Treatment

**Figure 2:**
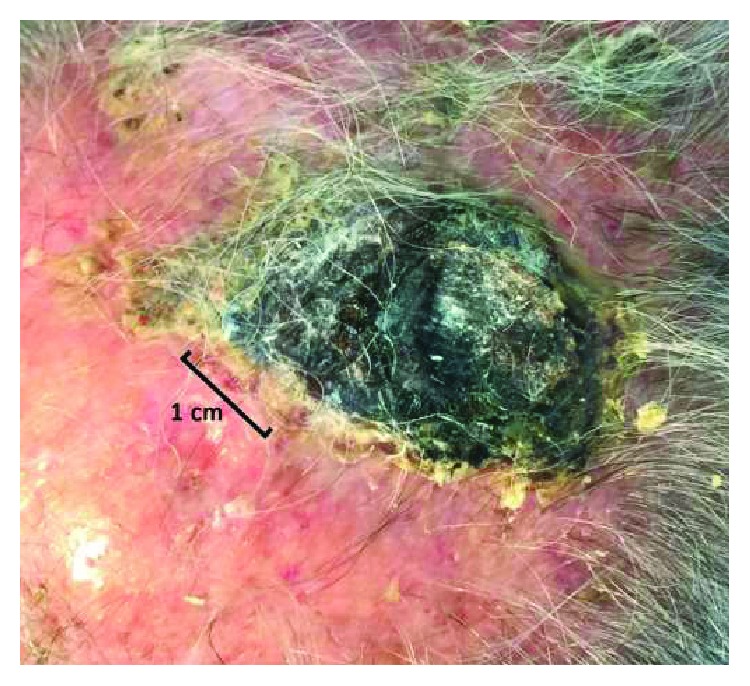
Eschar after ECT treatment.

**Figure 3:**
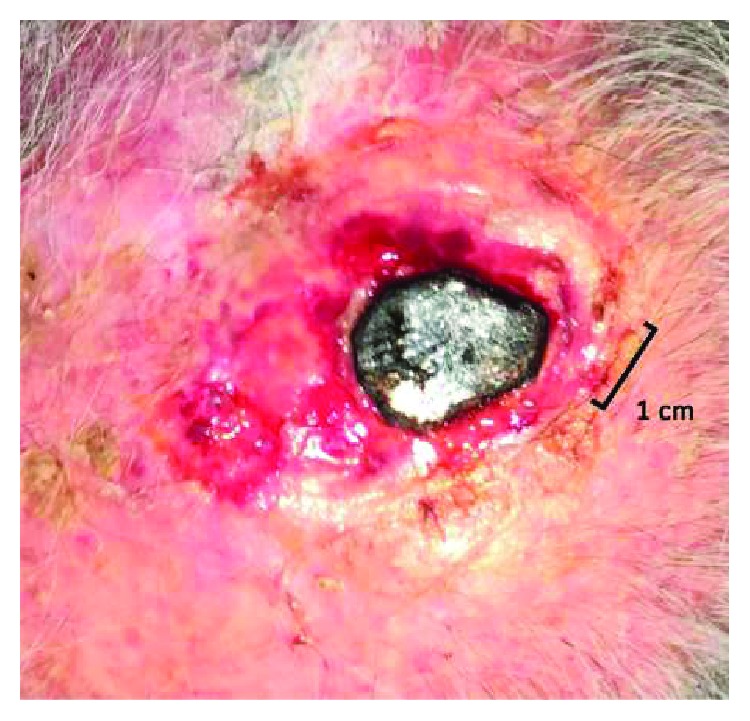
Eschar detached.

**Figure 4:**
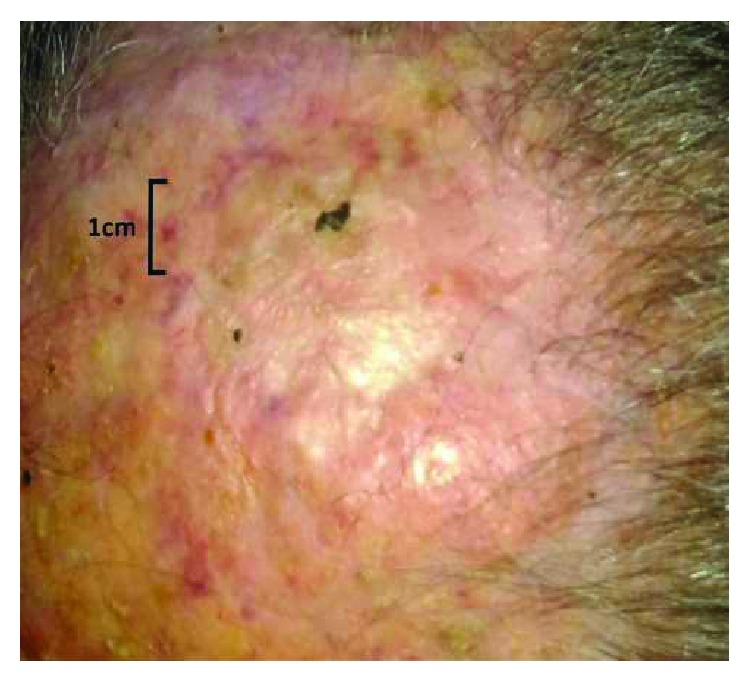
Restitutio ad integrum.

A local treatment with ECT has been performed in the region of the cutaneous relapse using a Cliniporator® EPS-02 produced by IGEA®. The procedure is reported following the guidelines by Campana et al. [9]. Electroporation was performed after 8 minutes from the end of slow bleomycin intravenous infusion (Bleomycin TEVA, 28500 IU-15000 IU/m^2^ of body surface area, diluted in 100 cc of physiologic solution in 15 minutes). The patient underwent a general anesthesia (deep sedation with Propofol—Diprivan®) before an adjustable linear needle electrode by IGEA*®* was introduced into the tumor mass at a depth of 15 mm; also, a safety margin of 1 cm was treated around it. A series of 8 pulses of 1000 V/cm was delivered at a frequency of 5 kHz and duration of 100 microseconds, as recommended by ESOPE guidelines [10]. In order to guarantee that the tumor received a sufficient amount of bleomycin, the treatment was completed within 15 minutes after the end of the infusion. A sterile plate medication was finally applied on the treatment site. The patient did not report residual pain and did not have any kind of complication. Four weeks after ECT treatment, the mass diminished in diameter and appeared completely necrotized (Figure 2). Eight weeks after treatment, all that remained was an eschar that detached, leaving an erythematous zone of reepithelialization (Figure 3). This area disappeared within three months, with a complete restitutio ad integrum (Figure 4).

## 3. Discussion

Atypical fibroxanthoma (AFX) is a tumor correlated to sun exposure that most commonly presents as a solitary red or pink papule or dermal ulcerative nodule of the head or neck of elderly people [11]. AFX affects males more frequently (76%) and it is associated with different risk factors, in particular xeroderma pigmentosum, Li-Fraumeni syndrome, and immunosuppression [12]. In particular, it has been demonstrated that AFX occurs more frequently in transplanted patients. It rarely metastasizes and infrequently recurs, with a rate of 7-10% [11, 13]. However, making a definitive diagnosis of AFX is challenging, and frequently, it is hard to distinguish from pleomorphic dermal sarcoma (PDS), previously known as undifferentiated pleomorphic sarcoma, squamous cell carcinoma (SCC), or malignant melanoma [12, 14, 15]. Head and neck localization represents 90% of the total, while the other 10% appears on the limbs or on the trunk [12]. Histological characteristics and immunohistochemical markers are essential for the right diagnosis; for example, the absence of immunostaining for cytocheratins, S-100, and HMB45 is useful for excluding both SCC and malignant melanoma [14]. In fact, AFX is a dermal-arising lesion of fibrohistiocytic origin and it is considered to be similar to PDS, more precisely a superficial less aggressive variant, sharing genetic alterations, such as chromosome 9p and 13q deletions [11, 12]. Moreover, a study demonstrated that UV-induced p53 mutations and CCND1/CDK4 were essential in tumorigenesis of both AFX and PDS, supporting the theory that AFX is a superficial variant of PDS [16]. Furthermore, activating mutations in HRAS and PIK3CA were also observed in both tumor types, even if with a greater frequency of HRAS mutations in PDS than AFX [16, 17]. Interestingly, in all AFX and PDS tumors, overexpression of p53, CCND1, and CDK4 was found. Despite these findings, it is still not fully understood whether AFX and PDS tumors are related neoplasms representing the extremes of a “spectrum” or whether they are two different malignant entities [14]. Taking into account all these considerations, a second consultation was requested for our case, confirming the difficulty to have an established diagnosis. In fact, if SCC could be excluded because of CK negativity, the weak positivity for S-100 and HMB45 did not allow ruling out a melanocytic origin. Anyway, we believe it was most likely a case of fibrohistiocyte-arising neoplasm, probably PDS, given the presence of necrosis and the subcutaneous extension. There are no clear recommendations for the treatment of these tumors, but a surgical exeresis is considered the best option, and it was thus undertaken. After relapse, given the superficial localization and the positive results of previous studies, the patient was considered for an electrochemotherapy treatment.

ECT is a local antitumor therapy that started being developed in the 80s [18]. Starting from the first clinical study in 1990, ECT demonstrated to be highly effective, with complete response rates of 73% and objective response (complete and partial response) rates of 85%. Another prospective study on heterogeneous cutaneous neoplasm showed tumor response rate at 60 days of 88% (complete, 50%) [19]. More recently, two studies considering head and neck cancers were published by EURECA (European Research on Electrochemotherapy in Head and Neck Cancer). One of them focused on head and neck skin cancer and showed OR rates ranging from 59% to 100%. In addition, 1-year overall survival and local DFS rates (76% and 89%, respectively) were favorable [3]. From the other, focusing on mucosal recurrent head and neck cancers, it resulted that electrochemotherapy is practicable and effective in recurrent mucosal head and neck cancer with an overall objective response of 56% in intention-to-treat analysis [4]. In 7% of the patients, a long-term complete remission with no evidence of disease was observed [4]. The standardization of ECT was obtained during a multicentric study (European Standard Operating Procedures of Electrochemotherapy (ESOPE)) in 2006 [20] and updated in July 2018 [10]. As previously written, the chemotherapeutic agents most commonly used in association with ECT are bleomycin and cisplatin. In our case, we utilized intravenous bleomycin as more frequently executed for cutaneous tumors. In fact, it has been demonstrated that cell electroporation increases the amount of bleomycin entering the cells up to several thousand times, while the effect on intracellular concentration of cisplatin is less pronounced [21]. The reason of that finding must be searched in the different capability of the two drugs to permeate cells in normal condition. In fact, bleomycin is a completely nonpermeant drug that needs active carriers to be delivered throughout the cell membrane; on the contrary, cisplatin, a smaller and less hydrophilic molecule, is able to cross passively through the membrane so the increased permeability obtained by electroporation is extremely effective primarily with bleomycin. Bleomycin is a chemotherapeutic drug, whose main action is represented by DNA damaging and breaking, resulting in cell apoptosis. This mechanism mainly affects cycling cells, resulting in a selective killing of the neoplastic ones, preserving the healthy cells. The normal tissues located in proximity to the tumor are often infiltrated by neoplastic cells that can lead to disease relapse after an unpredictable period, and the classic tumor treatments for their aggressiveness do not treat margins extensively. Vice versa, considering the selectivity for tumor cells, with ECT, it is possible to treat safely large margins around the nodules with good results. In addition to the raised toxicity, ECT effectiveness also relies on other mechanisms. It determines transient local ischemia and vascular damage, reducing the blood flow by 80% and causing a drug entrapment in the tumor at the very time when the cell is permeable, by the so-called “vascular lock” [21, 22]. A study in 2012 performed with intravital microscopy demonstrated that after EP a consistent increase of permeability occurs in the blood vessels, while the flow is reduced because of a constriction of afferent arterioles [23]. Another recent study based on intravital microscopy demonstrated gap junction alterations as a result of EP application, stressing the key role of these structures in determining the increase of vessel permeability [24]. In the case of ECT, this can be used favorably. In this perspective, it is a fair assumption that this phenomenon would decrease drug washout and it must be considered for the drug administration timing. Furthermore, it has been demonstrated that vascular lock lasts longer in tumors than in the normal tissue and that the recovery of the initial blood flow takes hours [21, 22]. In fact, a long-lasting hypoxia in the area contributes to the high effectiveness of this treatment and is probably responsible for the hemorrhagic tumor bleeding stoppage [25]. Subsequently, the treatment of hemorrhagic and painful nodule, as occurred in our patient, is a very interesting indication for ECT. Moreover, it appears that ECT is capable to evoke an autoimmune response that is selective for neoplastic cells. In fact, the exposition of tumoral cells to EP alone determines the externalization of calreticulin, while the association with bleomycin provokes the exposition also of other damage-associated molecular profiles (DAMPs) as HMGB1 [26].

ECT use is approved for skin and subcutaneous tumors, independently of their histological origin [27]. However, it seems to be more efficient in the case of small, mildly aggressive, cutaneous primary or metastatic lesion. As previously described, cutaneous and subcutaneous cancers of different histological derivation have been successfully treated with ECT. For example, Macri et al. reported the case of a neck skin metastasis of oral SCC with a complete response and no signs of disease recurrence after two years [28]. Interestingly, similar results have been obtained also in two cases of dermatofibrosarcoma protuberans, confirming the versatility of this approach [29, 30]. On the contrary, there are only few studies considering PDS. Campana et al. published a phase II trial on soft tissue sarcomas with 8 cases of undifferentiated sarcoma and only one complete response, but there are no case reports or specific works on this kind of tumor [31].

## 4. Conclusions

To the best of our knowledge, no data concerning ECT as a locoregional treatment for AFX/PDS are available. Therefore, we present the first case in literature and hence the importance of this report in order to expand the application field of this new promising treatment also for AFX and PDS, considering its effectiveness and safety in elderly patients. However, further studies are needed to confirm our results.
